# Non-Restorative Low Anterior Resection Is Associated with Poor Intermediate-Term Oncological Outcomes in MRI-Defined Rectal Cancer

**DOI:** 10.3390/cancers17183074

**Published:** 2025-09-19

**Authors:** Ritch T. J. Geitenbeek, Mark Broekman, Thijs A. Burghgraef, Esther C. J. Consten, Roel Hompes

**Affiliations:** 1 Department of Surgery, Groningen University Medical Center, University of Groningen, 9713 GZ Groningen, The Netherlands; 2 Department of Surgery, Meander Medical Center, 3813 TZ Amersfoort, The Netherlands; 3 Department of Surgery, Jeroen Bosch Hospital, 5211 GZ ‘s-Hertogenbosch, The Netherlands; 4 Department of Surgery, Amsterdam University Medical Center, Location Amsterdam Medical Center, 1105 AZ Amsterdam, The Netherlands

**Keywords:** total mesorectal excision, rectal cancer, low anterior resection, local recurrence, disease-free survival, overall survival

## Abstract

Background: Non-restorative low anterior resection (NRLAR) may result in inferior oncological outcomes compared to restorative low anterior resection (RLAR) and abdominoperineal resection (APR). While NRLAR is often performed when poor functional or technical challenges are anticipated, comprehensive data on its oncological outcomes remain scarce. This study aimed to retrospectively evaluate the intermediate-term oncological outcomes of patients—who underwent RLAR, NRLAR, or APR for primary rectal cancer. Methods: This analysis included all elective NRLAR, RLAR, and APR procedures for primary rectal carcinoma performed across 11 Dutch centers from 2013 to 2020. The primary outcome was 3-year disease-free survival (DFS). Secondary outcomes included 3-year overall survival (OS) and 3-year local recurrence (LR). KaplanMeier survival analysis with log-rank testing and multivariate Cox regression analysis were employed. Results: A total of 253 (12.5%) patients underwent NRLAR, 1109 (55.0%) RLAR, and 656 (32.5%) APR. NRLAR was associated with a lower 3-year DFS (71.4%) versus RLAR (82.0%) and APR (77.4%) (*p* = 0.003). The 3-year OS was lower for NRLAR (82.9%) versus RLAR (93.5%) and APR (90.2%) (*p* < 0.001), with a higher 3-year LR rate for NRLAR (8.1%) versus RLAR (3.3%) and APR (4.5%) (*p* = 0.003). Multivariate Cox regression analyses confirmed NRLAR as an independent predictor for poorer DFS (HR 1.34; 95% CI: 1.01–1.80; *p* = 0.046), OS (HR 1.57; 95% CI: 1.04–2.36, *p* = 0.032), and higher LR risk (HR 2.66; 95% CI: 1.53–4.65; *p* <= 0.001). Conclusions: NRLAR is associated with poorer intermediate-term oncological outcomes. When technically feasible, restorative options should be considered, and prospective studies are required to further investigate causal relationships.

## Simple Summary

Non-restorative low anterior resection (NRLAR) may result in inferior oncological outcomes compared to restorative low anterior resection (RLAR) and abdominoperineal resection (APR) for the surgical treatment of rectal cancer. This study aimed to retrospectively evaluate the intermediate-term oncological outcomes of patients who underwent RLAR, NRLAR, or APR for primary rectal cancer. This analysis included all elective NRLAR, RLAR, and APR procedures for primary rectal carcinoma performed across 11 Dutch centers from 2013 to 2020. Multivariate Cox regression analyses confirmed NRLAR as an independent predictor for poorer DFS (HR 1.34; 95% CI: 1.01–1.80; *p* = 0.046), OS (HR 1.57; 95% CI: 1.04–2.36, *p* = 0.032), and higher LR risk (HR 2.66; 95% CI: 1.53–4.65; *p* <= 0.001). Therefore, NRLAR should be avoided when technically feasible alternatives exist.

## 1. Introduction

The cornerstone of surgical intervention in rectal cancer is radical surgery according to the total mesorectal excision (TME) principle [1,2]. While a restorative low anterior resection (RLAR) remains the most common procedure, sphincter preservation may be unattainable for either oncological or functional reasons. In these patients, an abdominoperineal resection (APR) is required [3]. A possible third approach constitutes a non-restorative low anterior resection; this involves cross-stapling of the rectal stump and constructing an end colostomy, commonly called an ultra-low Hartmann’s procedure [4].

In randomized controlled trials, NRLAR constitutes less than 5% of cases [5,6]. In the real world, in unselected patient populations—especially in northern Europe—NRLAR’s incidence can surge to as high as 25% of all low anterior resections [7,8,9,10]. Although sphincter preservation was feasible with acceptable oncological margins, the rationale for non-restorative surgery is usually unspecified and remains ill-defined. Unplanned NRLAR may unfold due to technical challenges encountered during pelvic dissection, such as a narrow pelvis, obesity, bulky tumors, or intricate anatomical configurations, especially in male patients [11,12].

While existing studies suggest an association between NRLAR and inferior oncological outcomes compared to APR and restorative surgery, the bulk of this evidence predates the advent of laparoscopic TME (L-TME), robot-assisted TME (R-TME), and transanal TME (TaTME) [13,14,15]. Moreover, studies that incorporate these techniques rely on relatively older data and often are hampered by limited sample size [15,16].

Therefore, the primary objective of this study is to elucidate the three-year oncological outcomes among a large cohort of rectal cancer patients undergoing NRLAR, RLAR, and APR within dedicated laparoscopic, robot-assisted, and transanal centers.

## 2. Materials and Methods

### 2.1. Study Design

This study was designed as a retrospective multicenter cohort analysis conducted by the Minimally Invasive Rectal Cancer Taskforce (MIRECA). The objective was to compare intermediate-term oncological outcomes after NRLAR, RLAR, and APR. The cohort has been previously described [17].

### 2.2. Study Population

All consecutive patients who underwent oncological rectal resection for MRI-defined primary rectal adenocarcinoma positioned at a distance of ≤12 cm of the anorectal junction (ARJ) between 1 January 2015, and 31 December 2020, were identified from eleven centers in the national Dutch ColoRectal Audit (DCRA) database. The DCRA is mandatory nationwide registry with prospective data collection and independent quality control. Each participating hospital was considered a dedicated high-volume unit, performing a minimum of 40 TME procedures annually [18,19].

Patients were excluded if they (1) underwent local excision only, (2) had prior transanal endoscopic microsurgery (TEM) before TME, (3) presented with metastatic disease (cM1), or (4) underwent surgery in a palliative or emergency context. The study protocol and reporting adhered to the STROBE guideline [20].

### 2.3. Outcomes and Definitions

The primary endpoint was 3-year disease-free survival (DFS). Secondary endpoints included 3-year overall survival (OS), 3-year local recurrence (LR) rate, and 3-year systemic recurrence (SR). Resection type was defined according to the procedure carried out during the index TME. Detailed definitions of all outcomes are provided in Supplementary File S1.

### 2.4. Treatment Approach and Follow-Up

All cases were evaluated by a multidisciplinary tumor board at the local institution. Treatment strategies, including neoadjuvant therapy, were decided in accordance with National guidelines [21]. Surgical resection consisted of a full TME, performed as either NRLAR, LAR, or APR depending on tumor location and patient-related factors. Postoperative follow-up was carried out according to national recommendations.

### 2.5. Statistical Analysis

Comparisons between groups were stratified by type of resection (NRLAR, RLAR, and APR). Categorical variables were tested with Fisher’s exact test. Continuous variables were compared with the Wilcoxon rank-sum test if not normally distributed and with Student’s *t*-test otherwise. Three-year oncological outcomes were estimated with Kaplan–Meier methods and compared using the log-rank test.

Multivariable Cox regression with backward selection (significance level 0.05) was applied to explore associations between resection type and oncological outcome (DFS, OS, LR, and SR). Variables were selected based on literature evidence and included age, sex, body mass index, ASA classification, distance of the inferior border of the tumor to the ARJ, neoadjuvant therapy, pathological TNM classification, CRM involvement, and the minimally invasive TME technique. Variance inflation factors (VIF) were calculated to check for multicollinearity. Multiple imputation was performed when data were missing at random. Statistical significance was defined as *p* < 0.05. All analyses were conducted in ‘R’ version 4.1.3 (R Foundation for Statistical Computing, Vienna, Austria).

## 3. Results

### 3.1. Baseline Characteristics

The MIRECA dataset documented 3049 TME procedures. Following the exclusion of all ineligible patients, 2018 patients were included in the analysis. Of these, 1109 (55.0%) underwent RLAR, 656 (32.5%) underwent APR, and 253 (12.5%) underwent NRLAR.

Patients in the NRLAR group were notably older (mean age 75.48 years (standard deviation (SD): 9.72), compared to 65.54 (SD: 9.74) in the RLAR group and 69.34 (SD: 11.03) in the APR group, respectively, *p* < 0.001). They also more frequently presented as ASA III classification (34.4%, compared to 14.0% in RLAR and 23.5% in APR, *p* < 0.001), had a higher incidence of previous abdominal surgery (37.2%, compared to 24.5% in RLAR and 29.7% in APR, *p* < 0.001), exhibited EMVI exceeding 5 mm on MRI more frequently (22.9%, compared to 13.9% in RLAR and 17.2% in APR, *p* < 0.001), and had a higher prevalence of cT3-stage (67.2%, compared to 57.2% in RLAR and 55.0% in APR, *p* < 0.001) (Table 1).

In the APR group, patients had a shorter distance of the inferior border of the tumor to the ARJ on MRI (1.50 cm [0.0, 3.2], versus 7.00 [5.0, 9.0] and 6.00 [3.5, 8.0] in RLAR and NRLAR, respectively, *p* < 0.001). They also more frequently displayed MRF involvement (39.3%, versus 19.4%, and 30.4% in RLAR and NRLAR, respectively, *p* < 0.001), had a higher incidence of cT4-stage (13.3%, versus 5.0%, and 5.5% in RLAR and NRLAR, respectively, *p* < 0.001), and more often received chemoradiotherapy (37.2%, versus 24.3%, and 24.3% in RLAR and NRLAR, respectively, *p* < 0.001).

### 3.2. Surgical Characteristics and Postoperative Outcomes

NRLAR was predominantly performed laparoscopically (46.6%), while RLAR and APR were more frequently performed using the robot-assisted technique (53.0% and 47.1%, respectively). Conversion rates were higher in the NRLAR group than in the RLAR and APR groups (8.3% versus 3.5% and 4.0%, respectively, *p* = 0.011). Intraoperative complications occurred more frequently in the APR group than in the RLAR and NRLAR groups (10.7% versus 4.2% versus 6.3%, respectively, *p* < 0.001) (Table 2).

### 3.3. Pathological Outcomes

Overall, pT-stages were higher in the NRLAR group (*p* = 0.005). The quality of the TME specimen was reported lowest in the APR group (*p* < 0.001). Pathological CRM involvement was 2.8% for RLAR, 7.3% for APR, and 4.2% for NRLAR (*p* <0.001). Perforations were observed in 1.0% of patients undergoing RLAR, 4.4% patients undergoing APR, and 2.0% patients undergoing NRLAR (*p* < 0.001) (Table 2).

### 3.4. Oncological Outcomes

Table 2 compares oncological outcomes. NRLAR demonstrated significantly inferior rates of 3-year DFS, 3-year OS, and 3-year LR.

**Figure 1:**
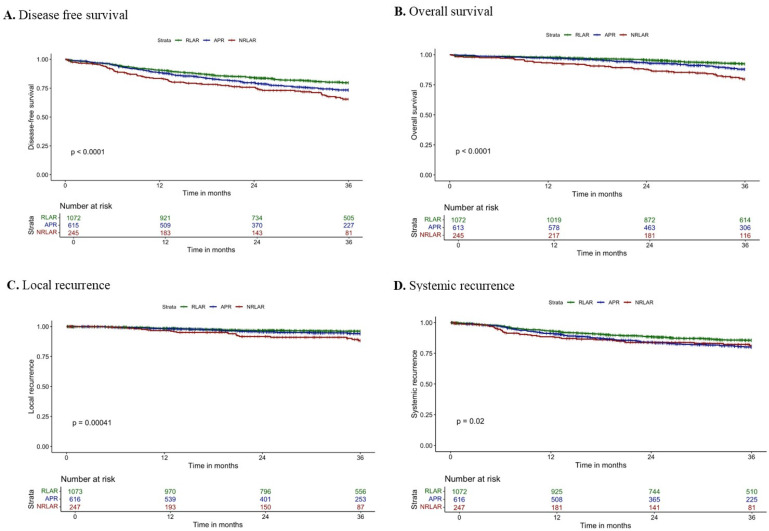
Kaplan–Meier survival curves of (**A**) 3-year disease-free survival, (**B**) 3-year overall survival, (**C**) 3-year local recurrence, and (**D**) 3-year systemic recurrence.

The 3-year DFS rate was 82.0% after RLAR, 77.4% after APR, and 71.4% after NRLAR (log-rank: *p* = 0.003; Figure 1A). Multivariable Cox regression analyses demonstrated that NRLAR was independently associated with worse 3-year DFS (HR 1.34; 95% CI: 1.01–1.80; *p* = 0.046) (Table 3). Other independent predictors for 3-year DFS included age >80, ASA class III/IV, chemoradiation, CRM involvement, pT4 stage, and pN1–2 stage.

The 3-year OS rate was 93.5% after RLAR, 90.2% after APR, and 82.9% after NRLAR (log-rank: *p* < 0.001; Figure 1B). Multivariable Cox regression analyses demonstrated that NRLAR was independently associated with worse 3-year OS (HR 1.57; 95% CI: 1.04–2.36, *p* = 0.032) (Table 3). Other independent predictors for 3-year OS included age >70, ASA class III/IV, chemoradiation, pT4 stage, and pN1–2 stage.

The 3-year LR rate was 3.3% after RLAR, 4.5% after APR, and 8.2% after NRLAR (log-rank: *p* = 0.003; Figure 1C). Multivariable Cox regression analyses demonstrated that NRLAR was independently associated with a higher 3-year LR rate (HR 2.66; 95% CI: 1.53–4.65; *p* <= 0.001) (Table 4). Other independent predictors for 3-year LR included CRM involvement, pT4-stage, and pN1–2 stage.

The 3-year SR rate was 12.5% after RLAR, 16.4% after APR, and 15.0% after NRLAR (log-rank: *p* = 0.077; Figure 1D). Multivariable Cox regression analyses demonstrated no independent association between resection type and 3-year LR rate (Table 4). Factors that were independently associated with 3-year SR included ASA class III/IV, radiotherapy, chemoradiation, pT4 stage, and pN1–2 stage.

## 4. Discussion

In this retrospective multicenter study encompassing 2018 patients diagnosed with rectal cancer across 11 dedicated centers in the Netherlands, NRLAR demonstrated inferior intermediate-term oncological outcomes compared to RLAR and APR. After correction for confounding, NRLAR emerged as an independent predictor for decreased 3-year DFS and OS, alongside higher rates of 3-year LR.

The observed 3-year DFS rates after RLAR (82.0%) and APR (77.4%) align with those reported in prominent randomized controlled trials (RCTs) comparing open TME with L-TME, such as the ALaCART, COLOR II, and ACOSOG Z6051 trials [22,23,24]. However, previous RCTs have frequently excluded NRLAR patients from comparative analyses, limiting the available literature. Two retrospective analyses conducted by Hol et al. and Roodbeen et al. did compare oncological outcomes following NRLAR [15,16]. In line with the observed 3-year DFS after NRLAR (71.4%) of the present study, Hol et al. reported a 3-year DFS rate (70.1%) and also found an independent association between NRLAR and inferior 3-year DFS [16]. Roodbeen et al. reported an even lower 3-year DFS rate for NRLAR (62.0%) but found no significant association between NRLAR and 3-year DFS in multivariate analysis [15]. This discrepancy may be attributed to factors beyond resection type, such as the relatively higher incidence of CRM involvement and elevated pT stages within their NRLAR cohort.

The 3-year OS rates observed following NRLAR (82.9%), RLAR (93.5%), and APR (90.2%) are consistent with previous studies [25,26,27]. Hol et al. and Roodbeen et al. reported similar 3-year OS rates and also demonstrated NRLAR’s independent association with inferior 3-year OS after adjusting for confounding factors [15,16]. Moreover, two older studies reporting data from 1995 to 2003 and 2006 to 2010 found lower overall OS rates across resection types. This disparity is likely due to older data and different treatment regimens at the time. However, both studies mirrored the current study’s trend, with the lowest OS rates for NRLAR [13,14].

The 3-year LR rates after RLAR (3.3%), APR (4.5%), and NRLAR (8.1%) align with previous studies reporting the long-term oncological outcomes of these resections [28,29]. Both Ortiz et al. and Hol et al. reported elevated 3-year LR rates after NRLAR and highlighted significantly higher rates of CRM positivity in the NRLAR group as a strong contributor to the higher 3-year LR rates [14,16]. In contrast to these findings, in the study by Roodbeen et al., positive CRM rates were comparable between resection types and could not explain the differences observed in LR rate and survival [15].

Regarding SR, multivariate analyses did not establish an independent association between NRLAR and higher SR rates compared to RLAR and APR. The observed 3-year SR rates (NRLAR: 15.0%, RLAR: 12.5%, APR: 16.4%) align with prior studies reporting on the long-term oncological outcomes of TME [28,30]. However, Hol et al. did find a significant association between NRLAR and SR, possibly due to a higher incidence of advanced cN status and variations in EMVI, a strong predictor for distant metastasis, which was not available in their dataset [16,31,32]. In the present multivariable analyses, neoadjuvant radiotherapy and chemoradiation were unexpectedly associated with worse SR rates. This finding likely reflects selection bias, where patients receiving neoadjuvant therapies often represent a cohort with more advanced or biologically aggressive disease, leading to higher recurrence rates despite intensive treatment. Interestingly, despite presenting with more advanced disease, patients in the NRLAR group received neoadjuvant chemoradiation at similar rates to RLAR patients and less frequently than APR patients. This likely reflects selection bias, as older and frailer patients were often considered unfit for multimodal therapy. While such undertreatment may have contributed to poorer outcomes, NRLAR remained independently associated with inferior DFS, OS, and LR after adjusting for neoadjuvant therapy, consistent with previous studies where treatment was equally administered across groups.

The independent relationship between NRLAR and DFS, OS, and LR is likely non-causal, as the mere construction of an anastomosis should not inherently influence oncological or survival outcomes, provided a proper oncological resection is performed. Given the multifactorial nature of cancer recurrence and survival, numerous factors must be considered when evaluating these outcomes after NRLAR.

NRLAR patients were inherently exposed to baseline risk factors such as advanced age, ASA III/IV classification, increased T stage, and higher EMVI prevalence on MRI, placing this group at a greater risk of recurrence or death. These factors were accounted for in the present multivariable Cox regression analysis, which demonstrated that NRLAR remained independently associated with poorer oncological outcomes after statistical correction for these confounders. While recognizing that not all potential confounding factors could be included due to the limited number of events, the results suggest that worse outcomes after NRLAR may not merely result from selective bias.

Although the dataset did not include specific information on pelvic sepsis, previous studies have suggested that this complication may contribute to the increased rates of LR observed in NRLAR patients. This phenomenon, often secondary to anastomotic leakage or rectal stump blow-out, has been associated with significant local inflammatory responses that may compromise oncological outcomes [33,34]. While anastomotic leakage in RLAR patients is often promptly detected and managed, delayed recognition and treatment of pelvic sepsis in NRLAR patients could exacerbate its impact on oncological and survival outcomes [35,36]. However, further research is needed to elucidate the specific role of pelvic sepsis in rectal cancer prognosis.

Disparities in neoadjuvant therapy administration may have influenced survival outcomes. NRLAR patients received neoadjuvant chemotherapy significantly less often than RLAR patients despite their unfavorable pathological profiles. This may be because the local multidisciplinary cancer board considered some NRLAR patients too frail for both a restorative procedure and neoadjuvant therapy, given their older age and higher ASA class. Studies in which neoadjuvant therapy was equally administered between groups observed similar associations with NRLAR, indicating that therapy differences alone do not fully explain these outcome variations [15,16]. Furthermore, neoadjuvant therapy guidelines changed drastically between the analyses performed by previous studies and the present study [2,37]. Adjuvant chemotherapy was administered in a limited number of patients (2.3% RLAR vs. 2.1% APR vs. 2.0% NRLAR), as it is not routinely provided in the Netherlands due to the uncertain oncological benefits in MRI-defined rectal tumors [38,39,40,41]. While adjuvant therapy is typically reserved for patients with unfavorable pathological risk factors, such as positive margins or high tumor grade, there was no significant difference in its administration between groups.

Other factors that warrant consideration include intraoperative technical challenges. The decision not to restore bowel continuity may have been made intraoperatively following difficult TME dissection, as indicated by the higher conversion rate for NRLAR patients. Along this line of thought, it should be considered that the ability to construct an anastomosis serves as a measure of surgical difficulty, which is potentially associated with poorer oncological and survival outcomes. Technical challenges during resection, as evidenced by the higher conversion rates in the NRLAR group, may reflect the complexity of pelvic dissection in this population. While conversions have been hypothesized to correlate with worse survival outcomes in previous literature, this association is largely attributed to incomplete resections or positive CRM involvement, both robust predictors for LR, DFS, and OS [42,43]. In this cohort, however, NRLAR demonstrated resection quality comparable to RLAR and superior to APR in terms of CRM positivity and TME specimen completeness. Therefore, while conversions in NRLAR likely reflect procedural difficulty, they are unlikely to have significantly influenced the oncological outcomes observed in this study. Strikingly, when examining all three resection types, APR reported the highest rates of incomplete and nearly complete TME specimens (8.8% and 23.0%, respectively), the highest rate of CRM involvement (7.3%) and perforation rate (4.4%). It must be acknowledged that APR is typically reserved for tumors located at or fixated to the anal sphincter or just above it. In such cases, the distal location of the tumor corresponds to a tapered mesorectum, which increases the technical complexity of achieving an R0 resection. Nevertheless, NRLAR still outperformed APR regarding oncological outcomes and survival, suggesting that the inferior outcomes after NRLAR likely stem from factors beyond TME specimen quality and CRM involvement alone.

This study is subject to several limitations that warrant acknowledgment. Importantly, the retrospective design introduces the risk of selection bias and limits the possibility of establishing causality. Despite the use of multivariable regression to account for key confounders, residual confounding cannot be ruled out. The unequal group sizes and the higher age and comorbidity burden in the NRLAR cohort reflect real-world surgical decision making, where non-restorative resection is more often performed in older and frailer patients. Although these baseline imbalances were adjusted for in the multivariable analyses, residual confounding may still be present. Although dedicated techniques were employed in expert centers specializing in L-TME, R-TME, or TaTME, variability in surgeon experience across hospitals could have influenced the decision between the types of resection. Furthermore, the present study included MRI-defined rectal tumors within 12 cm from the ARJ, as a recent study suggested that tumors between 12 and 15 cm from the ARJ might be classified as sigmoid tumors, should the novel sigmoidal take-off definition be employed [44]. Classifying tumors according to this definition was not feasible for the current study due to the inability to evaluate preoperative MRI scans in some centers. Another important limitation is that relevant confounders, such as the surgeon’s experience and intraoperative decision-making details, could not be fully accounted for. An additional key limitation of this study is the absence of functional and quality-of-life outcomes, restricting assessment of NRLAR’s benefit profile. As NRLAR aims to prevent severe functional impairments, focusing solely on oncological outcomes limits clinical applicability. Another limitation of this study is the absence of systematically documented reasons for selecting NRLAR over RLAR or APR. The choice for a non-restorative approach is often multifactorial, depending on patient frailty, intraoperative anatomy, and technical complexity, but these factors are rarely recorded in a standardized fashion. As a result, the clinical rationale for NRLAR could not be assessed in this cohort. Lastly, the decision to perform a non-restorative resection may reflect anticipation of procedural challenges, indicated by the higher conversion rate and intraoperative complications, introducing selection bias despite multivariable adjustments. The strengths of the present study lie in the large national cohort, the use of a prospective audited registry, and the consistency of high-volume expert centers, which help mitigate some of these inherent shortcomings. Nevertheless, these findings should be interpreted with caution, and prospective studies or randomized controlled trials are required to confirm the observed associations.

Despite these limitations, these findings carry significant clinical implications. This study underscores NRLAR’s independent association with worse oncological outcomes compared to RLAR and APR, corroborating previous reports. Given the absence of prospective studies or RCTs providing insight into NRLAR’s outcomes, avoidance of NRLAR for rectal cancer treatment is recommended.

### Future Perspectives

In scenarios where non-restorative resection is deemed necessary, removal of the rectal stump through intersphincteric APR (iAPR) should be pursued. Compared with NRLAR, iAPR may decrease the risk of residual mesorectum and potentially lower the incidence of diversion proctitis and pelvic sepsis [45]. However, caution is warranted as iAPR is technically demanding and requires substantial experience and subspecialized training. Ongoing randomized trials comparing iAPR with NRLAR are expected to shed further light on their postoperative surgical morbidity [46].

Future research should not only focus on oncological safety but also systematically incorporate patient-reported outcomes and functional endpoints. The rationale for considering a non-restorative resection is often to avoid the severe functional sequelae of a very low anastomosis, such as low anterior resection syndrome, urgency, or incontinence. Therefore, a comprehensive evaluation of surgical strategies must balance oncological outcomes with quality of life and functional preservation.

Ongoing prospective studies, such as the nationwide VANTAGE trial, are specifically designed to address this gap by integrating oncological, functional, and quality-of-life outcomes into a single framework. These data will be essential to guide truly patient-centered decision making and to clarify the role of NRLAR compared to restorative and abdominoperineal approaches.

## 5. Conclusions

In this multicenter cohort of 2018 patients with MRI-defined rectal cancer, NRLAR was associated with significantly poorer oncological outcomes compared with RLAR and APR. Three-year disease-free survival was 71.4% after NRLAR versus 82.0% after RLAR and 77.4% after APR (*p* = 0.003), while overall survival was 82.9% for NRLAR compared to 93.5% and 90.2%, respectively (*p* < 0.001). Local recurrence was more than doubled after NRLAR (8.1%) compared to RLAR (3.3%) and APR (4.5%) (*p* = 0.003). After multivariable adjustment, NRLAR remained an independent predictor of inferior DFS (HR 1.34), OS (HR 1.57), and higher LR risk (HR 2.66). While this association cannot be interpreted as causal, it likely reflects a combination of patient frailty, technical challenges during low pelvic dissection, and the potential impact of pelvic sepsis. These findings highlight the need for careful surgical decision making and underscore the importance of prospective studies to further clarify causal mechanisms and optimize outcomes in this patient group.

## Figures and Tables

**Table 1:** Patient and tumor characteristics.

	RLAR	APR	NRLAR	
*n*	1109	656	253	*p*
**Patient and tumor characteristics**				
Sex (*n*, %)				0.095
Male	688 (62.0)	435 (66.3)	151 (59.7)	
Female	421 (38.0)	221 (33.7)	102 (40.3)	
Age (mean (SD))	65.54 (9.74)	69.34 (11.03)	75.48 (9.72)	<0.001
BMI (mean (SD))	26.15 (4.07)	26.51 (4.29)	26.73 (5.05)	0.066
ASA (*n*, %)				<0.001
I	251 (22.6)	101 (15.4)	27 (10.7)	
II	701 (63.2)	395 (60.2)	131 (51.8)	
III	155 (14.0)	154 (23.5)	87 (34.4)	
IV	2 (0.2)	6 (0.9)	8 (3.2)	
History of abdominal surgery (*n*, %)	272 (24.5)	195 (29.7)	94 (37.2)	<0.001
Distance to ARJ on MRI(median [IQR])	7.00 [5.00, 9.00]	1.50 [0.00, 3.20]	6.00 [3.50, 8.00]	<0.001
MRF involvement (*n*, %)				<0.001
No	806 (72.7)	352 (53.7)	161 (63.6)	
Yes	215 (19.4)	258 (39.3)	77 (30.4)	
Missing	88 (7.9)	46 (7.0)	15 (5.9)	
EMVI on MRI (*n*, %)				<0.001
None	326 (29.4)	141 (21.5)	48 (19.0)	
<=5 mm	330 (29.8)	182 (27.7)	74 (29.2)	
>5 mm	154 (13.9)	113 (17.2)	58 (22.9)	
Missing	299 (27.0)	220 (33.5)	73 (28.9)	
cT-stage (*n*, %)				<0.001
I	50 (4.5)	15 (2.3)	7 (2.8)	
II	355 (32.0)	190 (29.0)	61 (24.1)	
III	634 (57.2)	361 (55.0)	170 (67.2)	
IV	56 (5.0)	87 (13.3)	14 (5.5)	
Missing	14 (1.3)	3 (0.5)	1 (0.4)	
cN-stage (*n*, %)				<0.001
0	448 (40.4)	288 (43.9)	94 (37.2)	
I	292 (26.3)	192 (29.3)	69 (27.3)	
II	176 (15.9)	137 (20.9)	53 (20.9)	
Missing	193 (17.4)	39 (5.9)	37 (14.6)	
Neoadjuvant therapy (*n*, %)				<0.001
None	527 (47.5)	236 (36.0)	101 (39.9)	
SCRT	307 (26.7)	169 (24.5)	86 (34.0)	
LCRT	3 (0.3)	4 (0.6)	4 (1.6)	
CRT	269 (24.3)	244 (37.2)	62 (24.3)	
Missing	2 (0.2)	0 (0.0)	0 (0.0)	
Adjuvant chemotherapy (*n*, %)				
No	681 (61.4)	442 (67.4)	115 (45.5)	<0.790
Yes	26 (2.3)	14 (2.1)	5 (2.0)	
Missing	402 (36.2)	200 (30.5)	133 (52.6)	

*ARJ*, anorectal junction; *ASA*, American Society of Anesthesiology; *APR,* abdominoperineal resection; *BMI,* body mass index; *CRT*, chemoradiotherapy; *EMVI*, extramural vascular invasion; *IQR,* interquartile range; *LCRT*, long-course radiotherapy; *MRF,* mesorectal fascia; *MRI,* magnetic resonance imaging; *n*, number; *NRLAR*, non-restorative low anterior resection; *RLAR*, restorative low anterior resection; *SCRT*, short-course radiotherapy; *SD*, standard deviation.

**Table 2:** Surgical outcomes.

	RLAR	APR	NRLAR	
*n*	1109	656	253	*p*
Surgical technique (*n*, %)				
O-TME	17 (1.5)	25 (3.8)	11 (4.3)	<0.001
L-TME	346 (31.2)	295 (45.0)	118 (46.6)	
R-TME	588 (53.0)	309 (47.1)	87 (34.4)	
TaTME	158 (14.2)	25 (3.8)	37 (14.6)	
Other	0 (0.0)	2 (0.3)	0 (0.0)	
Operating time (mean (SD))	194.05 (69.51)	198.10 (66.15)	166.52 (71.52)	<0.001
Conversions (*n*, %)	39 (3.5)	26 (4.0)	21 (8.3)	0.011
Intraoperative complications (*n*, %)	47 (4.2)	70 (10.7)	16 (6.3)	<0.001
Bleeding (*n*, %)	1 (0.1)	7 (1.1)	0 (0.0)	0.004
Perforation (*n*, %)	23 (2.1)	22 (3.4)	7 (2.8)	0.255
Postoperative complications (*n*, %)	507 (45.7)	284 (43.3)	116 (45.8)	0.828
Anastomotic leakage (*n*, %)	162 (14.6)	0 (0.0)	0 (0.0)	<0.001
Clavien Dindo (*n*, %)				0.105
I	114 (10.3)	78 (11.9)	17 (6.7)	
II	185 (16.7)	95 (14.5)	56 (22.1)	
IIIa	27 (2.4)	17 (2.6)	7 (2.8)	
IIIb	136 (12.3)	66 (10.1)	22 (8.7)	
IVa	23 (2.1)	15 (2.3)	5 (2.0)	
IVb	10 (0.9)	4 (0.6)	2 (0.8)	
V	9 (0.8)	3 (0.5)	5 (2.0)	
				
**Pathological outcomes**				
pT-stage (*n*, %)				0.005
0	73 (6.6)	65 (9.9)	12 (4.7)	
I	145 (13.1)	55 (8.4)	29 (11.5)	
II	383 (34.5)	255 (38.9)	80 (31.6)	
III	482 (43.5)	263 (40.1)	120 (47.4)	
IV	22 (2.0)	16 (2.4)	10 (4.0)	
Missing	3 (0.3)	1 (0.2)	1 (0.4)	
pN-stage (*n*, %)				0.727
0	754 (68.0)	458 (69.8)	168 (66.4)	
I	254 (22.9)	144 (22.0)	66 (26.1)	
II	88 (7.9)	49 (7.5)	18 (7.1)	
Missing	13 (1.2)	5 (0.8)	1 (0.4)	
Mesorectum (*n*, %)				<0.001
Complete	888 (80.1)	437 (66.6)	185 (73.1)	
Nearly complete	159 (14.3)	151 (23.0)	54 (21.3)	
Incomplete	35 (3.2)	58 (8.8)	10 (4.0)	
Missing	27 (2.4)	10 (1.5)	4 (1.6)	
*p*CRM involvement (*n*, %)	29 (2.8)	43 (7.3)	10 (4.2)	<0.001
Perforation (*n*, %)	11 (1.0)	29 (4.4)	5 (2.0)	<0.001
				
**Oncological outcomes**				
Follow-up in months (median [IQR])	36.92 [24.30, 50.00]	31.90 [18.95, 45.98]	30.23 [17.11, 43.85]	<0.001
3-year local recurrence (%)	35 (3.3)	28 (4.5)	20 (8.1)	0.003
3-year systemic recurrence (%)	134 (12.5)	101 (16.4)	37 (15.0)	0.077
3-year disease-free survival (%)	879 (82.0)	476 (77.4)	175 (71.4)	0.003
3-year overall survival (%)	1002 (93.5)	553 (90.2)	203 (82.9)	<0.001

*APR,* abdominoperineal resection; *L-TME*, laparoscopic total mesorectal excision; *n*, number; *NRLAR*, non-restorative low anterior resection; *O-TME*, open total mesorectal excision; *pCRM*, pathological circumferential resection margin; *R-TME*, robot-assisted total mesorectal excision; *RLAR*, restorative low anterior resection; *SD*, standard deviation; *TaTME*, transanal total mesorectal excision.

**Table 3:** Multivariable Cox regression analysis for disease-free survival and overall survival.

	Disease-Free Survival				Overall Survival			
	Univariable		Multivariable		Univariable		Multivariable	
	HR (95% CI)	*p*-value	HR (95% CI)	*p*-value	HR (95% CI)	*p*-value	HR (95% CI)	*p*-value
Resection								
RLAR	Reference		Reference		Reference		Reference	
APR	1.34 (1.08–1.67)	0.008	1.08 (0.85–1.35)	0.540	1.57 (1.11–2.22)	0.010	1.07 (0.74–1.54)	0.726
NRLAR	1.81 (1.38–2.38)	<0.001	1.34 (1.01–1.80)	0.046	2.84 (1.94–4.16)	<0.001	1.57 (1.04–2.36)	0.032
Technique								
L-TME	Reference				Reference			
R-TME	0.92 (0.74–1.15)	0.473			0.86 (0.62–1.21)	0.391		
TaTME	1.11 (0.81–1.52)	0.506			1.22 (0.77–1.94)	0.391		
Sex								
Female	Reference				Reference			
Male	1.15 (0.94–1.42)	0.177			1.19 (0.86–1.63)	0.297		
Age								
<70	Reference		Reference		Reference		Reference	
70–80	1.29 (1.03–1.61)	0.026	1.15 (0.91–1.45)	0.233	2.27 (1.58–3.24)	<0.001	1.85 (1.28–2.72)	0.001
>80	1.89 (1.46–2.45)	<0.001	1.50 (1.13–1.99)	0.005	3.76 (2.57–5.49)	<0.001	2.66 (1.75–4.04)	<0.001
BMI								
<18.5	Reference				Reference			
18.5–25	0.73 (0.36–1.49)	0.386			1.46 (0.36–5.94)	0.598		
25–30	0.62 (0.31–1.27)	0.192			1.07 (0.26–4.39)	0.921		
>30	0.78 (0.31–1.61)	0.498			1.41 (0.34–5.86)	0.640		
ASA								
I/II	Reference		Reference		Reference		Reference	
III/IV	1.99 (1.61–2.46)	<0.001	1.78 (1.42–2.22)	<0.001	3.02 (2.23–4.10)	<0.001	2.27 (1.65–3.14)	<0.001
Distance to ARJ on MRI								
<5 cm	Reference				Reference			
>5 cm	1.15 (0.95–1.40)	0.153			1.25 (0.93–1.69)	0.142		
Neoadjuvant therapy								
None	Reference		Reference		Reference		Reference	
Radiotherapy	1.55 (1.21–1.97)	0.004	1.27 (0.99–1.63)	0.057	1.70 (1.18–2.44)	0.004	1.43 (0.99–2.07)	0.06
Chemoradiation	1.59 (1.26–2.02)	<0.001	1.59 (1.25–2.04)	<0.001	1.43 (0.98–2.08)	0.006	1.62 (1.10–2.38)	0.015
pT								
T0-T3	Reference		Reference		Reference		Reference	
T4	3.94 (2.63–5.90)	<0.001	2.82 (1.86–4.26)	<0.001	3.64 (2.02–6.55)	<0.001	2.86 (1.57–5.20)	<0.001
pN								
N0	Reference		Reference		Reference		Reference	
N1–2	2.84 (2.33–3.45)	<0.001	2.53 (2.07–3.09)	<0.001	1.95 (1.44–2.63)	<0.001	1.68 (1.23–2.29)	0.001
CRM								
Not involved	Reference		Reference		Reference		Reference	
Involved	2.49 (1.79–3.45)	<0.001	1.63 (1.16–2.29)	0.005	2.31 (1.42–3.77)	<0.001	1.61 (0.97–2.68)	0.066

*ARJ*, anorectal junction; *ASA*, American Society of Anesthesiology, *APR*, abdominoperineal resection; *BMI*, body mass index; *CI*, confidence interval; *CRM*, circumferential resection margin; *HR*, hazard ratio; *L-TME*, laparoscopic total mesorectal excision; *MRI*, magnetic resonance imaging; *NRLAR*, non-restorative low anterior resection; *R-TME*, robot-assisted total mesorectal excision *RLAR*, restorative low anterior resection; *TaTME*, transanal total mesorectal excision.

**Table 4:** Multivariable Cox regression analysis for local recurrence and systemic recurrence.

	Local Recurrence				Systemic Recurrence			
	Univariable		Multivariable		Univariable		Multivariable	
	HR (95% CI)	*p*-value	HR (95% CI)	*p*-value	HR (95% CI)	*p*-value	HR (95% CI)	*p*-value
Resection								
RLAR	Reference		Reference		Reference		Reference	
APR	1.50 (0.91–2.46)	0.112	1.38 (0.83–2.28)	0.214	1.41 (1.09–1.82)	0.010	1.24 (0.95–1.65)	0.120
NRLAR	2.91 (1.68–5.04)	<0.001	2.66 (1.53–4.65)	<0.001	1.40 (0.97–2.01)	0.071	1.22 (0.84–1.76)	0.302
Technique								
L-TME	Reference				Reference			
R-TME	0.80 (0.50–1.29)	0.364			0.97 (0.74–1.26)	0.805		
TaTME	1.01 (0.51–1.99)	0.980			1.11 (0.76–1.63)	0.596		
Sex								
Female	Reference				Reference			
Male	1.57 (0.97–2.56)	0.069			1.01 (0.79–1.29)	0.947		
Age								
<70	Reference				Reference			
70–80	1.09 (0.67–1.76)	0.727			1.10 (0.85–1.44)	0.468		
>80	1.10 (0.57–2.12)	0.777			1.26 (0.89–1.78)	0.196		
BMI								
<18.5	Reference				Reference			
18.5–25	*	*			0.54 (0.25–1.16)	0.112		
25–30	*	*			0.47 (0.22–1.01)	0.053		
>30	*	*			0.66 (0.30–1.45)	0.302		
ASA								
I/II	Reference				Reference		Reference	
III/IV	1.25 (0.74–2.10)	0.407			1.49 (1.13–1.96)	0.005	1.48 (1.13–1.96)	0.007
Distance to ARJ on MRI								
<5 cm	Reference				Reference			
>5 cm	1.50 (0.96–2.32)	0.073			1.06 (0.34–1.34)	0.641		
Neoadjuvant therapy								
None	Reference				Reference		Reference	
Radiotherapy	0.58 (0.32–1.08)	0.085			1.83 (1.34–2.50)	<0.001	1.44 (1.05–1.97)	0.023
Chemoradiation	1.22 (0.76–1.95)	0.420			2.19 (1.63–2.93)	<0.001	2.03 (1.51–2.74)	<0.001
pT								
T0-T3	Reference		Reference		Reference		Reference	
T4	5.33 (2.57–11.06)	<0.001	3.56 (1.65–7.66)	0.001	4.41 (2.83–6.89)	<0.001	3.08 (1.95–4.86)	<0.001
pN								
N0	Reference		Reference		Reference		Reference	
N1–2	1.82 (1.21–2.81)	0.006	1.63 (1.05–2.53)	0.031	4.10 (3.21–5.23)	<0.001	3.69 (2.88–4.74)	<0.001
CRM								
Not involved	Reference		Reference		Reference		Reference	
Involved	3.26 (1.73–6.15)	<0.001	2.25 (1.14–4.41)	0.019	2.41 (1.62–3.58)	<0.001	1.33 (0.88–2.02)	0.174

*ARJ*, anorectal junction; *ASA*, American Society of Anesthesiology, *APR*, abdominoperineal resection; *BMI*, body mass index; *CI*, confidence interval; *CRM*, circumferential resection margin; *HR*, hazard ratio; *L-TME*, laparoscopic total mesorectal excision; *MRI*, magnetic resonance imaging; *NRLAR*, non-restorative low anterior resection; *R-TME*, robot-assisted total mesorectal excision; *RLAR*, restorative low anterior resection; *TaTME*, transanal total mesorectal excision. * BMI could not be included in the multivariate analyses as local recurrence was not present in all BMI subgroups due to low overall incidence of local recurrence in the cohort.

## Data Availability

Data will be made available upon reasonable request by the corresponding author.

## Supplementary Materials

The following supporting information can be downloaded at: https://www.mdpi.com/article/10.3390/cancers17183074/s1, File S1.
