# Supplementary material for: Non-Restorative Low Anterior Resection Is Associated with Poor Intermediate-Term Oncological Outcomes in MRI-Defined Rectal Cancer
Source: Cancers (Basel). 2025 Sep 19;17(18):3074. doi: 10.3390/cancers17183074 (PMC12468712; doi:10.3390/cancers17183074)
Supplement: Supplementary file 1 [file cancers-17-03074-s001.zip › cancers-3820514-supplementary.pdf]

Supplementary materials

Supplementary methods

Baseline characteristics included age in years, body mass index (BMI), American Society of Anaesthesiologists (ASA) classification, history of abdominal surgery, distance to the anorectal junction (ARJ) on MRI in centimetres, mesorectal fascia (MRF) involvement on pre-treatment MRI, clinical TNM stage and administration of neoadjuvant therapy.

Conversion was defined as conversion to laparotomy to complete the mesorectal dissection. Surgical complications until 30 days postoperatively were categorized according to the Clavien-Dindo classification [1]. Clavien-Dindo grade III or higher was defined as major morbidity. Anastomotic leakage was defined as radiological or clinical evidence of anastomotic dehiscence according to the International Study group of Rectal Cancer grading and definition within 3 years following TME [2]. A pelvic abscess was defined as a collection visible on radiologic evaluation within 3 years following TME. Pelvic sepsis was defined as the occurrence of either a pelvic abscess or anastomotic leakage within 3 years following TME.

All surgical specimens were assessed and reported in accordance with the American Joint Committee on Cancer tumour-node-metastasis classification [3]. Pathological TNM stage, circumferential resection margin (CRM), tumour differentiation, tumour perforations, tumour deposits, lymphovascular invasion and quality of the mesorectum were reported. A positive CRM was defined as a tumour within 1 mm of the resection margin. Quality of the mesorectum was assessed using a three-tiered classification according to Quirke et al, consisting of complete, nearly complete, or incomplete resection [4].

References

1 Dindo D, Demartines N, Clavien PA. Classification of surgical complications: A new proposal with evaluation in a cohort of 6336 patients and results of a survey. Ann Surg. 2004;240:205–13.

2 Rahbari NN, Weitz J, Hohenberger W, *et al.* Definition and grading of anastomotic leakage following anterior resection of the rectum: A proposal by the International Study Group of Rectal Cancer. *Surgery*. 2010;147:339–51. doi: 10.1016/j.surg.2009.10.012

3 Amin MB, Edge SB, Greene FL, *et al. American Joint Committee on Cancer (AJCC). AJCC Cancer Staging Manual*. 2017.

4 Nagtegaal ID, Van de Velde CJH, Van Der Worp E, *et al.* Macroscopic evaluation of rectal cancer resection specimen: Clinical significance of the pathologist in quality control. *Journal of Clinical Oncology*. 2002;20:1729–34. doi: 10.1200/JCO.2002.07.010
